# Population-based sero-epidemiological investigation of the dynamics of SARS-CoV-2 infections in the Greater Accra Region of Ghana

**DOI:** 10.1038/s41598-022-25598-0

**Published:** 2022-12-14

**Authors:** Benedicta Ayiedu Mensah, Ignatius Cheng Ndong, Peter Kojo Quashie, Emilande Guichet, Benjamin Abuaku, Yaw Effah-Baafi, Kesego Tapela, Kwame Asiedu, Sekyibea Nana Ama Appiedu-Addo, Louisa Baaba Obbeng, Jones Amo Amponsah, Kwadwo Asamoah Kusi, Michael Ofori, Ahidjo Ayouba, David Courtin, Rachida Tahar, Eric Delaporte, Gordon Awandare, Nicaise Tuikue Ndam

**Affiliations:** 1 Noguchi Memorial Institute for Medical Research, University of Ghana, Accra, Ghana; 2 West African Centre for Cell Biology of Infectious Pathogens, University of Ghana, Legon, Accra, Ghana; 3 TransVIHMI, University of Montpellier, IRD and Institut National de La Santé Et de La Recherche Médicale (INSERM), Montpellier, France; 4 MERIT, IRD, Université de Paris, 75006 Paris, France; 5 Department of Biochemistry, Catholic University of Cameroon, Bamenda, Cameroon

**Keywords:** Infection, Infectious diseases, Viral infection

## Abstract

The coronavirus disease 2019 (COVID-19) pandemic devastated countries worldwide, and resulted in a global shutdown. Not all infections are symptomatic and hence the extent of SARS-CoV-2 infection in the community is unknown. The paper presents the dynamics of the SARS-CoV-2 epidemic in the Greater Accra Metropolis, describing the evolution of seroprevalence through time and by age group. Three repeated independent population-based surveys at 6-week intervals were conducted in from November 2020 to July 2021. The global and by age-groups weighted seroprevalences were estimated and the risk factors for SARS-CoV-2 antibody seropositivity were assessed using logistic regression. The overall age-standardized SARS-CoV-2 antibody seroprevalence for both spike and nucleocapsid increased from 13.8% (95% CI 11.9, 16.1) in November 2020 to 39.6% (95% CI 34.8, 44.6) in July 2021. After controlling for gender, marital status, education level, and occupation, the older age group over 40 years had a higher odds of seropositivity than the younger age group (OR 3.0 [95% CI 1.1–8.5]) in the final survey. Pupils or students had 3.3-fold increased odds of seropositivity (OR 3.2 [95% CI 1.1–8.5]) compared to the unemployed. This study reinforces that, SARS-CoV-2 infections have been significantly higher than reported.

## Introduction

The severe acute respiratory syndrome coronavirus 2 (SARS-CoV-2) was detected for the first time in Wuhan, China in December 2019 and causes the disease known as the Corona virus disease (COVID-19)^1^. The pandemic is ravaging countries across the world, devastating economies and led to a global lockdown from early 2020 to middle of 2021. The total number of cases worldwide is estimated at more than 570 million, with more than 6.4 million deaths^2^. To attempt solutions, a full understanding of the virus's serology and epidemiology is needed. However, the extent of spread of SARS-CoV-2 in the community is not clear since not all the infections are symptomatic^3^.

Ghana is one of the hardest hit countries in West Africa, with an estimated total of 168,000 confirmed cases, 1450 deaths, and as of August, 2022, a total of 18,520,455 vaccine doses have been administered^4^. While symptomatic case detection is going on in different testing centers across Ghana, any attempt to target asymptomatic SARS-CoV-2 cases could be useful to further paint a clearer picture of the pandemic in the country. There is therefore a need for robust testing tools and to strengthen the capacity to test. The detection and spread of an emerging respiratory pathogen are accompanied by uncertainties as to its main epidemiological and serological characteristics. In each country affected by the pandemic, the initial diagnostic capacity of the different laboratories was exceeded, causing surveillance activities to be focused mainly on patients with severe forms of the disease. This has also stretched our health system.

The number of patients who are or have been infected with the disease is currently unknown, since only symptomatic cases are being tested routinely. Information on the proportion of infections with little or no symptoms and their role in human-to-human transmission is incomplete and discordant. While the World Health Organisation (WHO) report following its initial visit to China highlighted a few asymptomatic cases, the early results of an Italian investigation where the entire village population was screened by PCR for SARS-CoV-2 showed that half of the infected individuals had no symptoms. We do not know if these patients were asymptomatic or pre-symptomatic, meaning they had no symptoms at the time of the test but developed symptoms thereafter^5^. In 2020 the dynamics of the pandemic and the impact of the interventions were exclusively simulated by models, which could be improved by data from the population providing information on the level of group immunity.

The tests presently used to diagnose COVID-19 are molecular tests for the detection of the SARS-CoV2 genome by RT-PCR, which allows an acute phase diagnosis of COVID-19. Serological tests on the other hand allow the detection of immunoglobulins produced by the body and directed against the virus. These tests help determine if a person has triggered an immune response to the virus and whether that lasts after the infection is over^6^. The production of IgM would be detectable from 7 days after infection and that of IgG from the 3rd week of infection, or even earlier. At the time of our study, there were no evidence demonstrating protective immunity of these antibodies^6,7^. The WHO recommends that countries carry out repeated population-based seroprevalence surveys to measure the dynamics of the epidemic^8^. These studies are even more important in sub-Saharan Africa, where access to care and PCR diagnostic tests is limited and where the younger population may be more likely to be asymptomatic in the event of infection.

This study set out to conduct a year-long population monitoring of SARS-CoV-2 in a sero-epidemiological survey in Accra. The study used a Luminex-based multiplex method developed for diagnosing various illnesses. The test can differentiate SARS-CoV-2 from SARS-CoV-1, MERS-COV and other coronaviruses prevalent in Africa and can also be reformulated to distinguish between recent (IgM) and previous (IgG) infections. This study conducted three 6-week spaced population surveys to measure the extent and dynamics of the SARS-CoV-2 pandemic in Accra, Ghana.

## Results

### Baseline characteristics of enrolled participants

Between November, 2020 and July, 2021, three consecutive surveys were conducted in November, March and July which enrolled 1236, 1013 and 1346 individual household members respectively across 4 districts in Accra Ghana. A final sample size of 3575 individuals in the study included consenting participants from 419, 465, 451 different households in survey 1, 2 and 3 respectively, making a total of 1335 (98.6%) out of the 1354 households visited (See full study profile in Supplementary Figures. S1, S2 and S3). The distribution of baseline characteristics is shown in Table 1. In summary, majority of study participants for each of the 3 surveys were females (59.3%, 57.4%, 50.1%), aged between 20 and 39 years (35.1%, 39.8%, 44.4%), were single (60.9%, 53.6%, 58.2%), had secondary education (46.8%, 52.5%, 53.0%) and were civil servants (29.5%, 29.5%, 26.8%).

**Table 1:** Baseline characteristics of study participants enrolled.

Baseline characteristics	Survey = 1, n (%)	Survey = 2, n (%)	Survey = 3, n (%)
	N = 1238	N = 1013	N = 1346
**Age group**			
≤ 19 years	434 (35.3)	239 (23.6)	330 (24.6)
20–39 years	431 (35.1)	403 (39.8)	595 (44.4)
≥ 40 years	363 (29.6)	371 (36.6)	415 (31.0)
**Gender**			
Male	486 (40.7)	429 (42.6)	654 (49.1)
Female	708 (59.3)	577 (57.4)	678 (50.9)
**Marital status**			
Single	753 (60.9)	542 (53.6)	783 (58.2)
Married/in union	379 (30.7)	384 (38.0)	472 (35.1)
Widowed	60 (4.9)	55 (5.4)	55 (4.1)
Divorced /separated	44 (3.6)	30 (3.0)	37 (2.7)
**Education level**			
Never attended school	132 (10.7)	106 (10.5)	112 (8.3)
Primary	319 (25.8)	211 (20.8)	308 (22.9)
Secondary	579 (46.8)	532 (52.5)	710 (52.9)
Tertiary	207 (16.7)	164 (16.2)	213 (15.9)
**Occupation**			
Unemployed	305 (24.7)	134 (13.2)	208 (15.4)
Civil servant	364 (29.5)	299 (29.5)	362 (26.8)
Student	282 (22.8)	258 (25.5)	339 (25.1)
Artisan	96 (7.8)	36 (3.6)	132 (9.8)
Other	188 (15.2)	286 (28.2)	311 (23.0)

### Crude and adjusted seroprevalence

**Figure 1:**
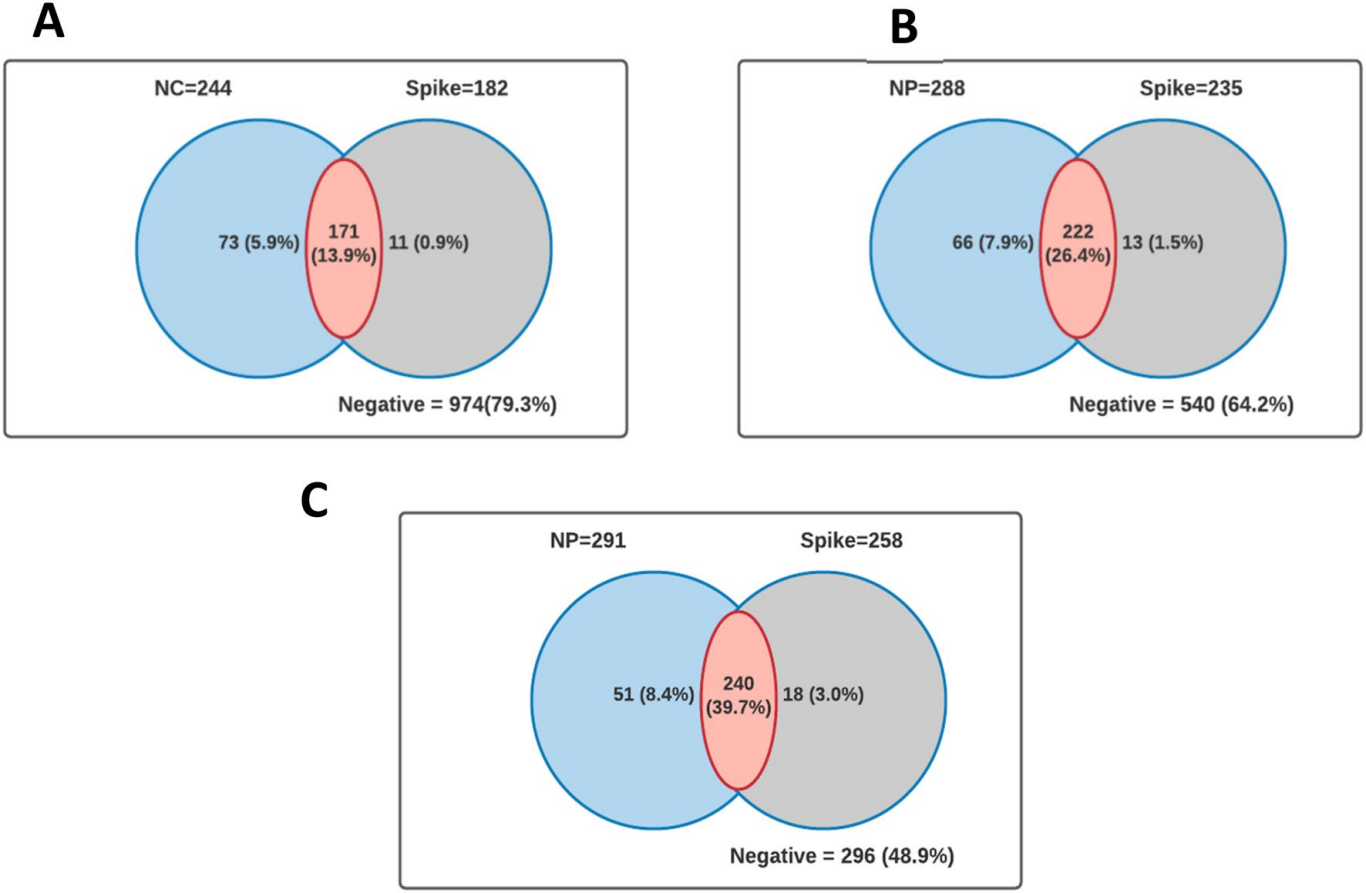
Venn diagram showing the distribution of seroprevalence by antigenic protein (**A**) survey 1, (**B**) survey 2, (**C**) survey 3.

**Figure 2:**
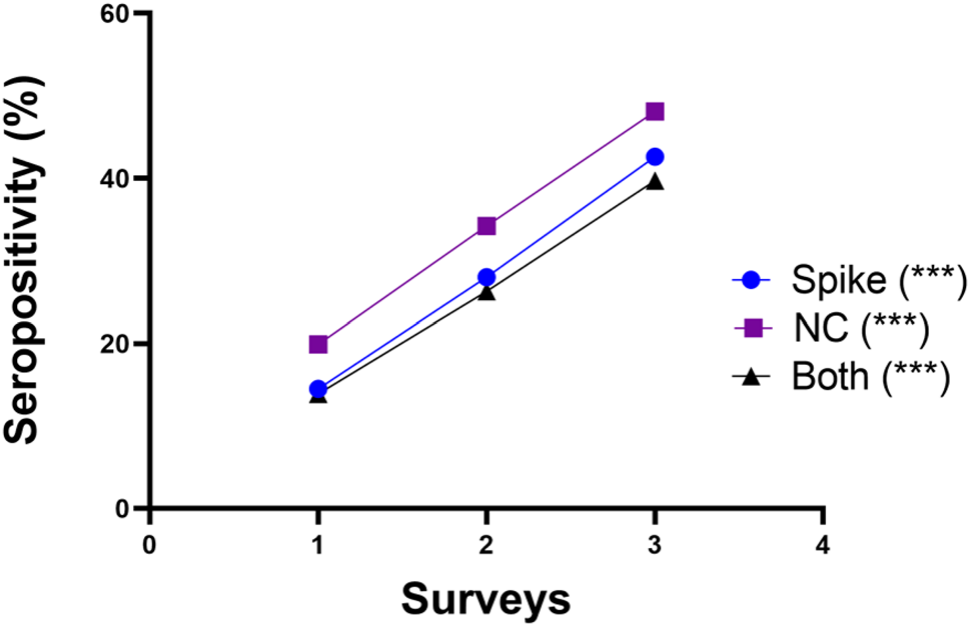
Trend plot of seropositivity for both spike and NC between November 2020 and July 2021. ***P < 0.001 (Cochran–Armitage test for trend).

The distribution of crude and age-standardized SARS-CoV-2 seroprevalence is shown in Figs. 1 and 2 and Table 2 respectively. The estimated crude seroprevalence of spike (SP) antibody was 14.8% in November 2020 and increased to 42.7% by July 2021. Also, the crude seroprevalence based on nucleocapsid (NC) surged from 19.8% in November 2020 to 48.1% in July 2021 (Fig. 1). The seropositivity for both SP and NC was similar to the trends observed for SP only (Fig. 2). The age-adjusted SARS-CoV-2 seroprevalence for both SP and NC increased from 13.8% (95% CI 11.9, 16.1) in November 2020 to 39.6% (95% CI 34.8, 44.6) in July 2021 (Table 1).

**Table 2:** Univariate analysis showing association of symptoms with sero-positivity.

Characteristics	Survey 1		Survey 2		Survey2	
	Seroprevalence age-weighted (%)	*P* value	Seroprevalence age-weighted (%)	*P* value	Seroprevalence age-weighted (%)	*P* value
**Total study population**	13.8 (11.9, 16.1)		25.7 (22.4, 29.3)		39.6 (34.8, 44.6)	
**Age group (years)**		0.622		**0.021**		0.731
0–19	13.5 (10.5, 17.2)		25.6 (19.9, 32.4)		38.6 (30.5, 47.3)	
20–39	13.3 (10.3, 17.1)		21.8 (17.5, 26.7)		41.8 (35.2, 48.7)	
40 and above	15.6 (12.1, 19.8)		31.6 (26.6, 37.2)		38.7 (32.2, 45.6)	
**Gender**		0.106		**0.026**		0.502
Males	0.11.5 (8.9, 14.8)		22.4 (18.2, 27.2)		40.9 (35.3, 46.8)	
Females	0.14.9 (12.4, 17.8)		29.5 (25.5, 33.9)		38.2 (32.1, 44.7)	
**Marital status**		0.088		**0.011**		0.985
Single	14.3 (11.9, 17.1)		21.6 (17.9, 25.8)		40.1 (34.7, 45.8)	
Married/union	11.8 (8.8, 15.6)		31.9 (26.9, 37.4)		39.3 (32.9, 46.2)	
Widowed	16.7 (9, 28.8)		27.1 (16.1, 41.8)		39.1 (21.1, 60.8)	
Divorced/separated	25.6 (14.5, 41.1)		36 (19.5, 56.7)		43.8 (21.9, 68.3)	
**Education level**		0.275		0.38		0.347
Never been to school	8.6 (4.7, 15.1)		23.2 (15.5, 33)		50.9 (36.9, 64.9)	
Primary	14.3 (10.8, 18.7)		28.6 (22.2, 35.9)		37.4 (29.3, 46.3)	
Secondary	15.3 (12.5, 18.6)		27.7 (23.6, 32.2)		38.9 (33.5, 44.6)	
Tertiary	13.5 (9.4, 19.1)		21.3 (15, 29.3)		39.8 (30.5, 49.9)	
**Occupation**		0.196		0.453		0.484
None	12.8 (9.4, 17.3)		25.5 (18, 34.7)		38 (27.6, 49.6)	
Civil servant	16.7 (13, 21)		28.2 (22.9, 34.3)		44.3 (35.6, 53.4)	
Pupil/student	14.4 (10.7, 19.3)		24.3 (18.8, 30.8)		42.4 (34, 51.2)	
Artisan	7.3 (3.4, 14.9)		13.8 (5.1, 32.2)		34.2 (24.5, 45.4)	
Other	13.4 (9.1, 19.3)		28 (22.5, 34.3)		36.3 (28.3, 45.1)	
**Symptoms score**		0.511		0.643		0.096
No symptoms	13.1 (9.1, 18.3)		26.2 (21.9, 31.1)		40.5 (33.7, 47.6)	
One to two symptoms	15.8 (12.5, 19.8)		28 (22.6, 34.3)		36.3 (29.1, 44.1)	
3 to 5 symptoms	12.5 (9.4, 16.5)		23.2 (17.4, 30.2)		39.3 (31.6, 47.6)	
More than 5 symptoms	16.1 (10.5, 23.9)		30.4 (18.6, 45.6)		61.3 (41.2, 78.2)	
**Hospitalized for any of these symptoms**		**0.012**		0.518		0.224
Yes	33.3 (17.2- 54.6)		20.8 (8.7, 42.1)		51.6 (31.6, 71.1)	
No	14 (11.8- 16.5)		26.9 (22.9, 31.3)		39 (33.9, 44.3)	
**Number of HH members**		0.052		0.25		0.53
1–2	16.2 (11.8- 21.8)		23.1 (18.3, 28.8)		43.9 (34, 54.3)	
3–5	10.9 (8.4- 14.1)		26.6 (22.2, 31.5)		37.8 (31.9, 44.2)	
> 5	15.8 (12.9- 19.3)		30 (23.9, 36.8)		40.5 (33.9, 47.5)	
**Have you been vaccinated?**				0.956		
Yes			26.7 (17.6, 38.3			
No			26.4 (23.3, 29.7)			

Significant values are in bold.

**Figure 3:**
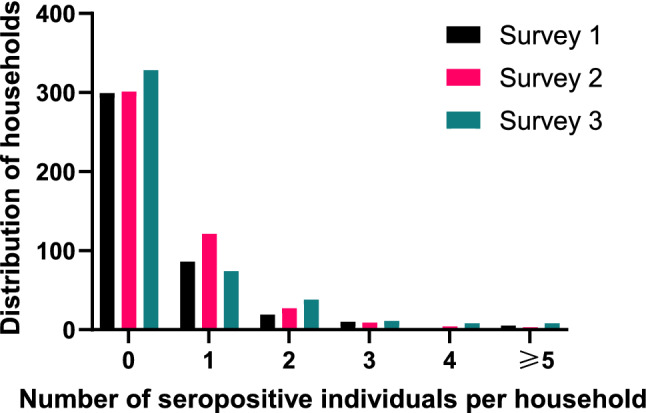
Distribution of households and number of individuals who were seropositive for both Spike and NC.

At the start of the survey, in November 2020, at the household (HH) level, the majority of households that reported seropositivity had only one SARS-CoV-2-positive member. In March 2021, the maximum number of household members who tested positive increased to four per HH. In July 2021, the number of HH with more than two infections per HH increased, slightly higher compared to the original two surveys, with some HH reporting as many as 5 or more infections. The detailed distribution of household seropositivity is reported in Fig. 3 and Supplementary Figure S4.

### Risk factors for seroprevalence

**Figure 4:**
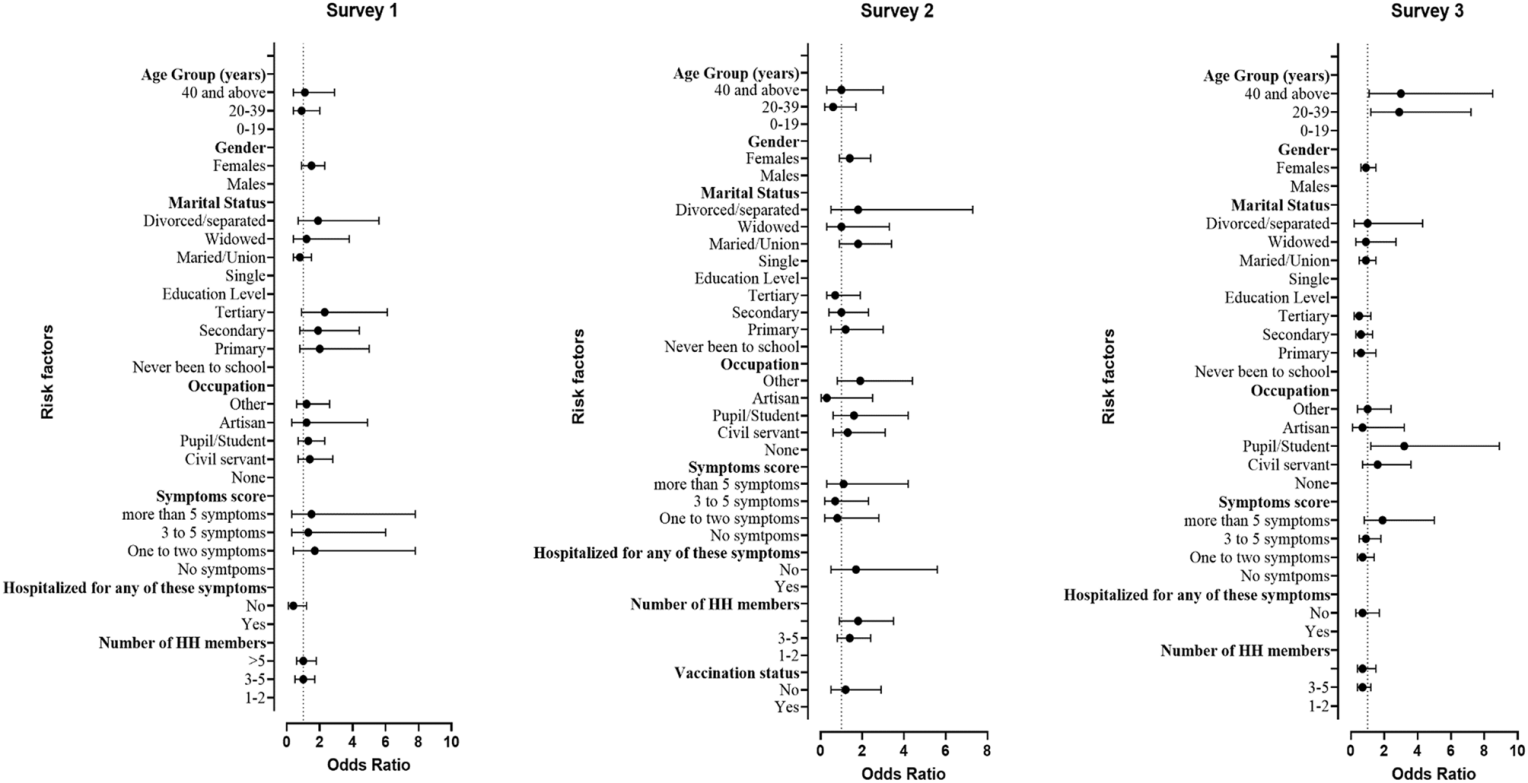
Forest plot of odds ratios of risk factors associated with Sars-Cov-2 seroprevalence in 3 consecutive surveys. Dots represents odds ratio and the bar is the confidence interval.

The univariate analysis of factors associated with age adjusted seroprevalence for participants who tested positive for both NC and SP revealed that having been hospitalized for any of the symptoms was a risk factor in survey 1 (Yes: 33.3%, % CI = [17.2–54.6], No: 14%, 95% CI = [11.8–16.5], p value = 0.012) shown in Table 2, but not in the multi-logistic regression (OR 0.1 [95% CI 0.1–1.2]) as shown in Fig. 4 and Supplementary Table 1. During the second survey, the univariate analysis revealed that age group, gender, and marital status all contributed to seropositivity risk (Table 2). Seropositivity was more prevalent in study participants aged 40 years and older (31.6%, 95% CI = [26.6, 37.2]), compared to the 0–19 years age group (25.6%, 95% CI = [19.9, 32.4]) and the 20–39 age group (21.8%, 95% CI = [17.5, 26.7]) (p = 0.021). Seropositivity was significantly higher (p = 0.026) in females (29.5%, 95% CI = [25.5, 33.9] than in males (22.4%, 95% CI = [18.2, 27.2]) and divorced participants (36%, 95% CI = [19.5, 56.7]) compared to single (21.6%, 95% CI = [17.9, 25.8]) and married participants (31.9%, 95% CI = [26.9, 37.4]) (p = 0.011). As illustrated in Fig. 4, age was not a significant factor in the multivariate analysis for survey 2. In the third and final survey conducted from June to July 2021, the multivariate logistic regression model showed older age and being educated were both significantly associated with seropositivity after adjusting for other variables. The odds of seropositivity was 3 times higher in those aged 20–39 years (OR 2.9 [95% CI 1.2–7.2]) or in the 40 years and above (OR 3.0 [95% CI 1.1–8.5]) compared to the 0–19 years. The odds of seropositivity was 3.2 times higher in the pupils, students or educated individuals (OR 3.2 [95% CI 1.1–8.5]) compared to the unemployed.

### Symptoms and health-seeking behaviour

Our study also explored which flu-like symptoms were associated with seropositivity in Ghana. Symptomatic infection (but not hospitalization) was significantly associated (P = 0.043) with seropositivity during the 1st survey in November 2020, but not in subsequent surveys (Table 3). Seropositivity was higher in participants who had more than 5 symptoms score (19/30 (63.3%)). Hospitalization for any symptom was only significantly associated with seropositivity in the final survey, in July 2021 (p = 0.008). Among participants hospitalized but not for flu-like symptoms, seropositivity was 33.3% (8/24 compared to 14% (124/887). Importantly, factors such as contact with a coronavirus/COVID-19 patient and previous PCR positive for SARS-CoV-2 infection, were not associated with SARS-CoV-2 antibody seropositivity (Table 3).

**Table 3:** Association of Flu symptoms with sero-positivity.

Symptoms	Survey = 1, n(%)	*P* value	Survey = 2, n(%)	*P* value	Survey = 3, n(%)	*P* value
**Symptoms score**		**0.043**		0.65		0.52
No symptom	87/214 (40.7)		98/374 (26.2)		29/222 (13.1)	
One to two symptoms	73/202 (36.1)		67/238 (28.2)		66/417 (15.8)	
3 to 5 symptoms	57/145 (39.3)		41/176 (23.3)		44/350 (12.6)	
More than 5 symptoms	19/30 (63.3)		14/46 (30.4)		20/124 (16.1)	
**Have you been hospitalized for any of these symptoms?**		0.17		0.58		**0.008**
Yes	16/31 (51.6)		5/23 (21.7)		8/24 (33.3)	
No	162/415 (39.0)		122/453 (26.9)		124/887 (14.0)	
**Have you been in contact with a coronavirus/COVID-19 patient?**		0.071		0.054		0.93
Yes	1/9 (11.1)		1/16 (6.3)		2/15 (13.3)	
No	228/558 (40.9)		183/652 (28.1)		132/934 (14.1)	
**Have you already been tested for coronavirus/COVID-19?**		0.39		0.90		0.53
Yes	9/28 (32.1)		19/73 (26.0)		6/54 (11.1)	
No	228/566 (40.3)		203/761 (26.7)		163/1155 (14.1)	
**What was the result of the test?**		0.13		0.87		0.24
Positive	2/2 (100.0)		1/4 (25.0)		1/2 (50.0)	
Negative	7/20 (35.0)		15/53 (28.3)		5/34 (14.7)	
Undetermined	0/3 (0.0)		0/1 (0.0)		0/2 (0.0)	
Never received result	0/2 (0.0)		1/7 (14.3)		0/11 (0.0)	
Did not get result	0/1 (0.0)		1/2 (50.0)		0/4 (0.0)	
Don’t know	0/0 (0.0)		1/6 (16.7)		0/0 (0.0)	

Significant values are in bold.

## Discussion

In Ghana, there are no official estimates of COVID-19 seroprevalence^4^. Given that the policy in Ghana during the period of study only offered free tests to symptomatic individuals^4^, and most paid tests were due to meet travel requirements, the full spectrum of SARS-CoV-2 exposure, including the extent and fraction of asymptomatic infections that do not require medical attention, needed to be investigated. Our findings indicate that seroprevalence at the three separate time points was always higher than the positivity reported at the time. This is not surprising, because whereas seropositivity is an indicator of cumulative exposure, infection testing is an indicator of disease incidence at any given time point^9^. Risk factors included older age groups and students in the final survey in 2021, when seroprevalence had increased from the initial 13–40%.

The seropositivity being measured in Ghana and West Africa is due to antibodies elicited by SARS-CoV-2, not other coronaviruses as erroneously assumed. The results of this study are credible because cross-reactivity due to the coronavirus (SARS-CoV-1) and the more distantly associated MERS-CoV was excluded^10^, any sample whose recorded SARS-CoV-2 seropositivity was lower than the average seropositivity of MERS-CoV or SARS-CoV-1 antigens was recorded as negative in our analysis^11^.

There was a rising trend in seroprevalence over the course of the eight-month study period, which is expected because, seroprevalence is a cumulative measure and an increase is usually indicative of ongoing transmissions. This pattern has also been demonstrated in a comparable Swiss study^12^ and a Chinese study^13^. This finding was not inconsistent with a previous study in Malawi^14^, Guinean^15^, Cameroon^16^ and another study in Ghana with different methodology^17^. That study, which used rapid diagnostic tests to detect SARS-CoV-2 seropositivity and screened individuals in public settings, discovered 19% seropositivity between August and December 2020 (in agreement with 19.3% for NC in survey 1), which rose to 25% by March 2021. That study however, is yet to be peer-reviewed. Our present study was more conservative in seroprevalence measurements, which strengthens the credibility of the 40% seropositivity recorded in June 2021. Our data also shows that cases detected during the acute phase of disease provide little information on the actual state of the outbreak^12^, and as such should not be the only information utilized for public health planning. The observed progression in the spread of the infection within the population did not result in an influx of patients into hospitals presenting serious symptoms requiring treatment probably due to the reduction of R0 of the first wave variant. Several reasons likely contributed to the relatively low seroprevalence of SARS-CoV-2 during the first survey, including government-imposed physical distancing guidelines or fewer individuals were infected at the time. These restrictions were eased over time, which may have led to the increase in seroprevalence as of April 2021^18^. These factors should be considered along with the general progression of the disease. The third-survey seroprevalence corroborated a previous meta-analysis of published data between April 2020-April 2021 which reported a combined seroprevalence of 25% (95% CI [13–39%]) in West Africa^19^.

Seroprevalence of SARS-CoV-2 antibodies significantly varied with age during the final survey after adjusting for other covariates like gender, occupation and number of household members. Seroprevalence increased with increasing age, incongruence with established COVID-19 literature. Since the pandemic's early stages, advanced age has been established as a significant predictor of risk of infection^20,21^, severe disease and poor outcomes^22^. Aside from that, it is unknown if the low number of cases in children is due to a milder disease course, with a higher proportion of asymptomatic cases, or to a decreased susceptibility to infection, as our results suggest^20–22^. This could be because ACE-2 expression levels change with age and asymptomatic/pauci-symptomatic infections cause a transient antibody response in younger age groups^21,23–26^. Low seropositivity in children in survey 1 was probably due to all schools being closed. Schools reopened in January 2021^27^. It is therefore note-worthy that during the final survey in July, pupils or students in school were more likely to be seropositive compared to the unemployed. This study potentially supports existing literature from other jurisdictions which found that high levels of mild or asymptomatic infection in school-going children, and that school attendance was an impediment to outbreak control^28^.

This study also explored if flu-like symptoms were associated with SARS-CoV-2 antibody seropositivity in Ghana. Flu-like symptoms were significantly associated with seropositivity during the first survey in November 2020. Consistent with studies conducted in China and Denmark^29,30^ where loss of taste or smell was the symptom most strongly associated with seropositivity. Surveys 2 and 3 showed no association between symptoms and seropositivity. Most individuals had not developed immunity to the virus at the time of survey 1, and initial spread may have been concentrated in people with increased susceptibility^31^. The high seropositivity recorded in surveys 2 and 3 supports asymptomatic and mild infections which would also affect this association. A review also provided an evidence of approximately 40–45% contribution of asymptomatic infection to SARS-CoV-2 infections which corroborates with our study^32^. The association of hospitalization observed in survey 3 is indicative of the circulating variants in Accra at the time of the study- B.1/B1.1 (Survey 1), Alpha/Eta (Survey 2) and Alpha/early Delta (Survey 3)^33^. Both Alpha and Delta were associated with sharp increases in disease severity in Ghana and during the Delta wave, hospitals were at maximum capacity^33^.

This study has real and maybe perceived weaknesses. The study was limited to the capital city Accra and may not be generalizable to the whole country. Accra had > 50% of all confirmed COVID-19 cases in Ghana throughout the sampling period, making it the most useful site for this study. By design, SARS-CoV-2 antibody seroprevalence could have been underestimated, both due to study design, as discussed above, or due to the kinetics of antibody seroconversion. Seroprevalence estimates represent infections at least two weeks (and up to 4–6 months) before sampling^13^. Thus, seroprevalence may have been underestimated since some participants may have recently been infected and not yet developed SP or NC response. Samples collected from infected individuals outside the time window of antibody response could produce false negatives. Due to the study's cross-sectional design, dynamic changes in antibody titer in infected patients over time were not studied. Long-term follow-up is needed to clarify the usefulness of these serology markers in estimating the cumulative attack rate^13^.

## Conclusions

This study reinforces the growing evidence that SARS-CoV-2 infections have been underestimated in Africa. More importantly, this study supports the hypothesis that SARS-CoV-2 infection in Africa has been mostly asymptomatic and mild. The sampling strategy, stringent methodology and sample size (~ 3% of all cumulative cases by survey 3) makes this study especially important. There are clearly non-social factors associated with seeming biological tolerance to COVID-19 infection in Ghana, which supports the reports from other African countries. This hypothesis can no longer be discounted and needs to be further explored.

## Methods

### Study area

The study site is Accra in the Greater Accra region of Ghana. The city covers an area of 225.7 km^2^ and an estimated population of 4.2 million people. Accra is divided into 29 administrative districts/municipalities/metropolis^34^ with a total of 5,423 Enumeration Areas (EAs) and 1,036,370 households, according to the 2010 census^35^. According to the 2017/2018 Ghana Living Standards Survey (GLSS7), Accra has 32.1% of its population under 15 years, 42.5% are from 15–39 years and 25.3% are 40+ years^36^.

### Study design, participants and sampling strategy

We conducted three cross-sectional population-based household surveys to assess SARS-CoV-2 prevalence in 4 districts of Ghana. The first was carried out in November 2020, second in March 2021 and the third and final survey in July 2021. These studies were carried out as part of the ARIACOV project and used the WHO population-based age stratified sero-epidemiological investigation protocol for COVID-19 infection version 2.0^8^. The study used multi-stage random sampling approach for sampling the participants of the study. Each study participant gave few (four) drops of blood on filter paper for SARS-CoV-2 serological analysis in the laboratory. This study was approved by the Institutional Review Board of Noguchi Memorial Institute for Medical Research (IRB approval no. 026/20-21) and the Ethics Committee of the Ghana Health Service (no. GHS-ERC011/08/20). Written informed consent was obtained from all adults to participate in the study and to be tested for SARS-CoV-2 antibodies. Written parental consent and child assent were obtained prior to enrollment of young participants (< 18 years). All methods were carried out in accordance with relevant guidelines and regulations.

### Sample collection and preparation

Dried blood spots (DBS) were collected on filter paper and stored with dessicant at 4 °C, until analyses. To extract plasma constituents from each DBS, a spot was cut out from the filter paper using 3 mm radius puncher (representing the equivalent of 10 µL of blood) and suspended into a well of a round bottom 96 well PCR containing 200 µL of dilution buffer (0.8 g NaH_2_PO_4_, 5 g Na_2_HPO_4_, 88 g NaCl, 1% BSA, 5% FBS, 0.2% Tween-20), making 20-fold dilution of the sample. The puncher was cleaned between samples by making two spots each on absorbent paper soaked in 70% alcohol followed by a blank filter paper. Each plate was then vortexed for 10 s to physically disrupt the filter paper, complement activity was deactivated in a heat block (56 °C) for 30 min followed by overnight incubation at 37 °C with shaking (250 rpm).

### Testing procedure

A Luminex-based multiplex system was used to detect IgG antibodies to nucleocapsid (NC) and spike (SP) of SARS-CoV-2, as previously described^11^. This dual target strategy for SARS-CoV-2 IgG antibody detection has a high sensitivity (100%) and specificity (99.7%)^11,37^. Results were expressed as the median fluorescence intensity (MFI) for 100 beads. Cutoff values were also previously determined using pre-COVID-19 samples and those from COVID-19 positive and hospitalized patients^38^. The assay included beads conjugated to NC and SP proteins of SARS-CoV-1, SARS-CoV-1 and MERS-CoV. Thus, each reading also detected cross-reactivity to NC and SP of both MERS-CoV and SARS-CoV-1. The average reading of SP and NC for MERS-CoV and SARS-CoV-1 are thus subtracted from each reading to eliminate positivity due to cross-reactive antibodies elicited by other coronaviruses. An experiment was validated when (1) the MFI value of the blank is less than 50 and (2) the MFI value of the negative control is less than 100. Samples were considered positive for SARS-CoV-2 SP signal if the signal exceeded 500 MFI and positive for SARS-CoV-2 NC if the reading exceeded 1000 MFI. A sample was considered positive for anti-SARS-CoV-2 IgG if positivity was established for both anti-SP IgG and anti-NP IgG.

### Statistical analyses

All Statistical analyses was performed using the statistical software STATA v15 and the significance level set at p < 0.05 for all tests. Weighted estimates of the SARS-CoV-2 antibody seroprevalence (and 95% confidence intervals) were calculated in the three surveys by age-standardizing the crude seroprevalence using available national census data. In each of the three surveys, the overall SARS-CoV-2 antibody seroprevalence was estimated and the seroprevalence stratified by sex and age group. The risk factors associated with positive anti-SARS-CoV-2 serology were determined using a weighted multivariable logistic regression.

## Supplementary Information

Supplementary Information.

## Data Availability

The datasets used and analyzed during the current study is available from the corresponding author on reasonable request.
