# Supplementary material for: Population-based sero-epidemiological investigation of the dynamics of SARS-CoV-2 infections in the Greater Accra Region of Ghana
Source: Sci Rep. 2022 Dec 14;12:21582. doi: 10.1038/s41598-022-25598-0 (PMC9748398; doi:10.1038/s41598-022-25598-0)
Supplement: Supplementary file 1 — Supplementary Information. [file 41598_2022_25598_MOESM1_ESM.docx]

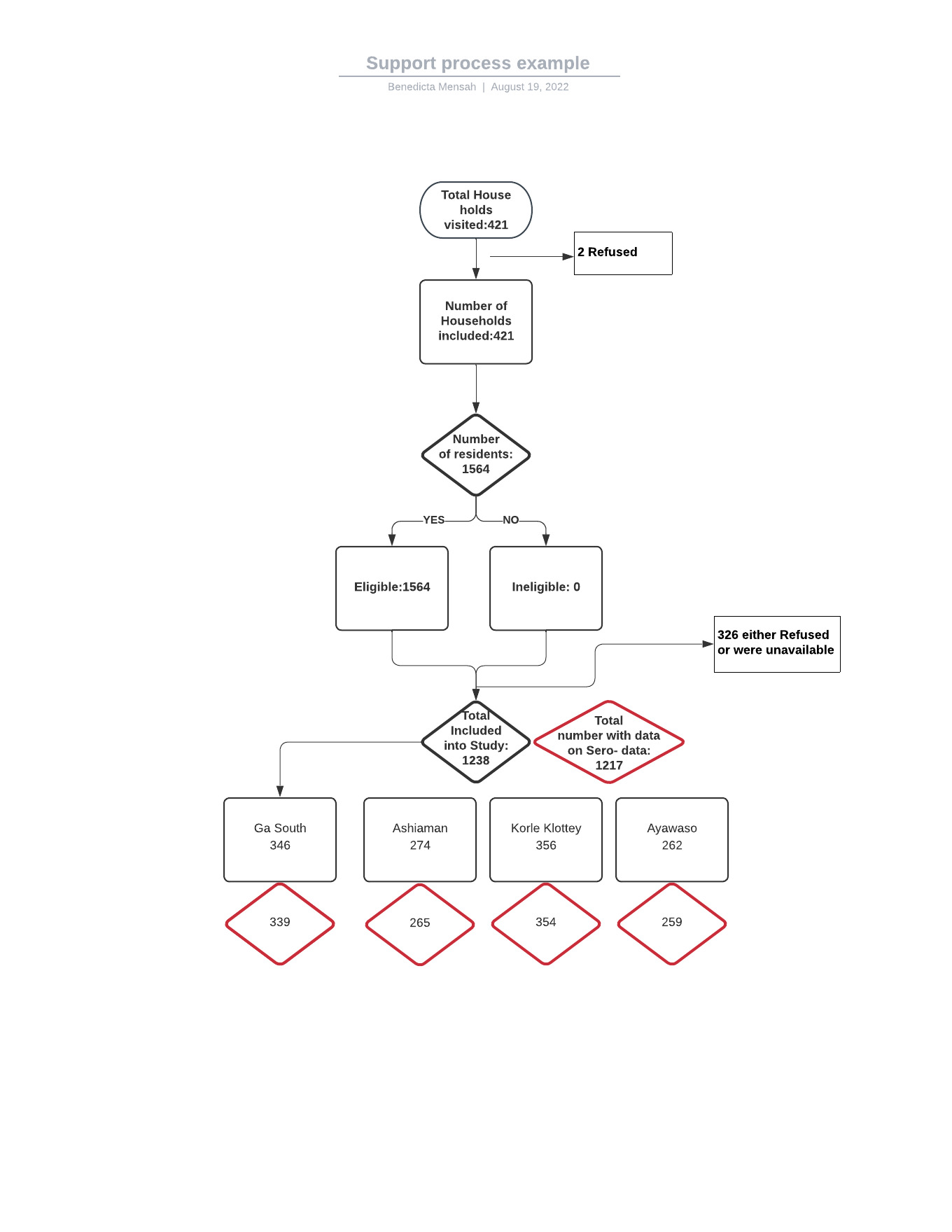


Figure S1. Description of the data for survey1


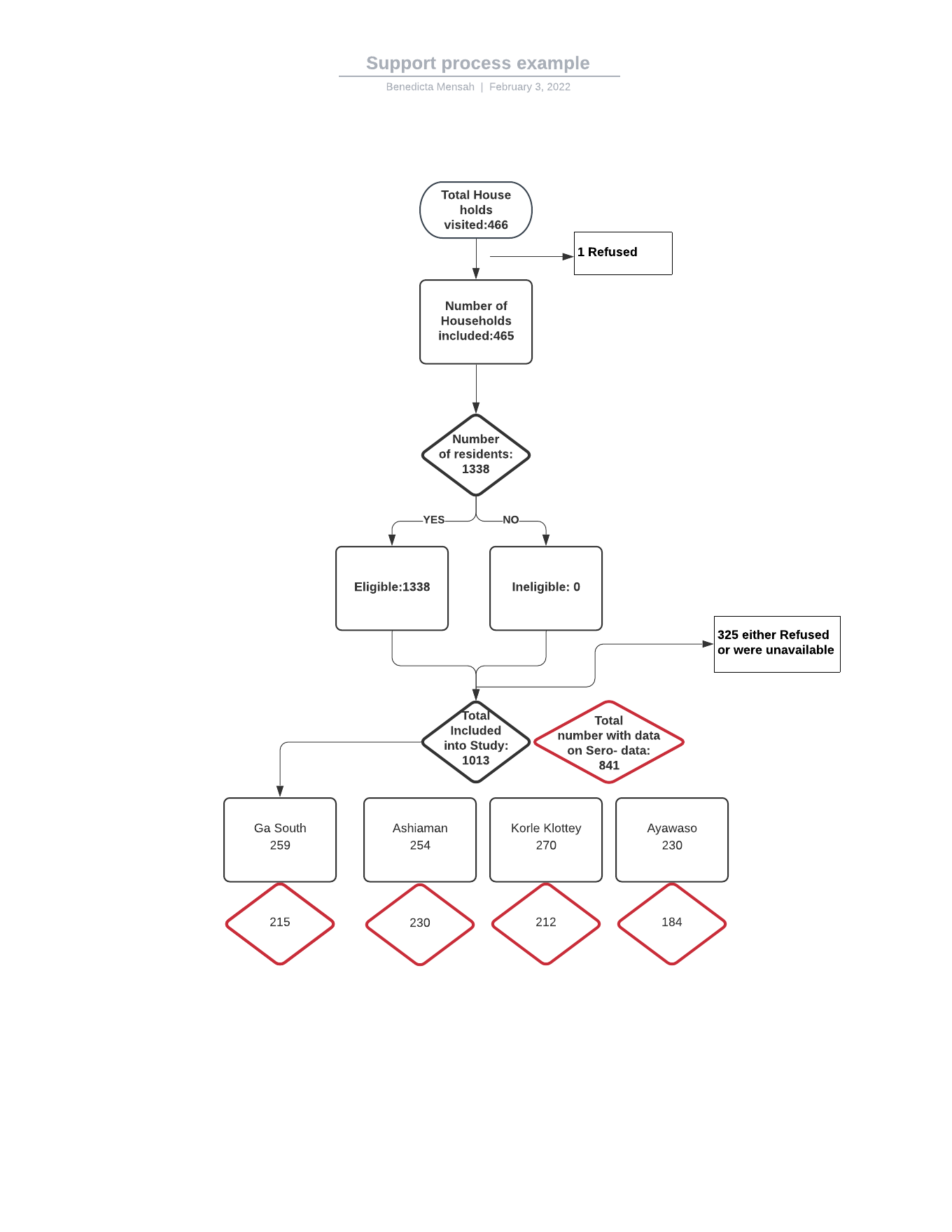


Figure S2. Description of the survey2


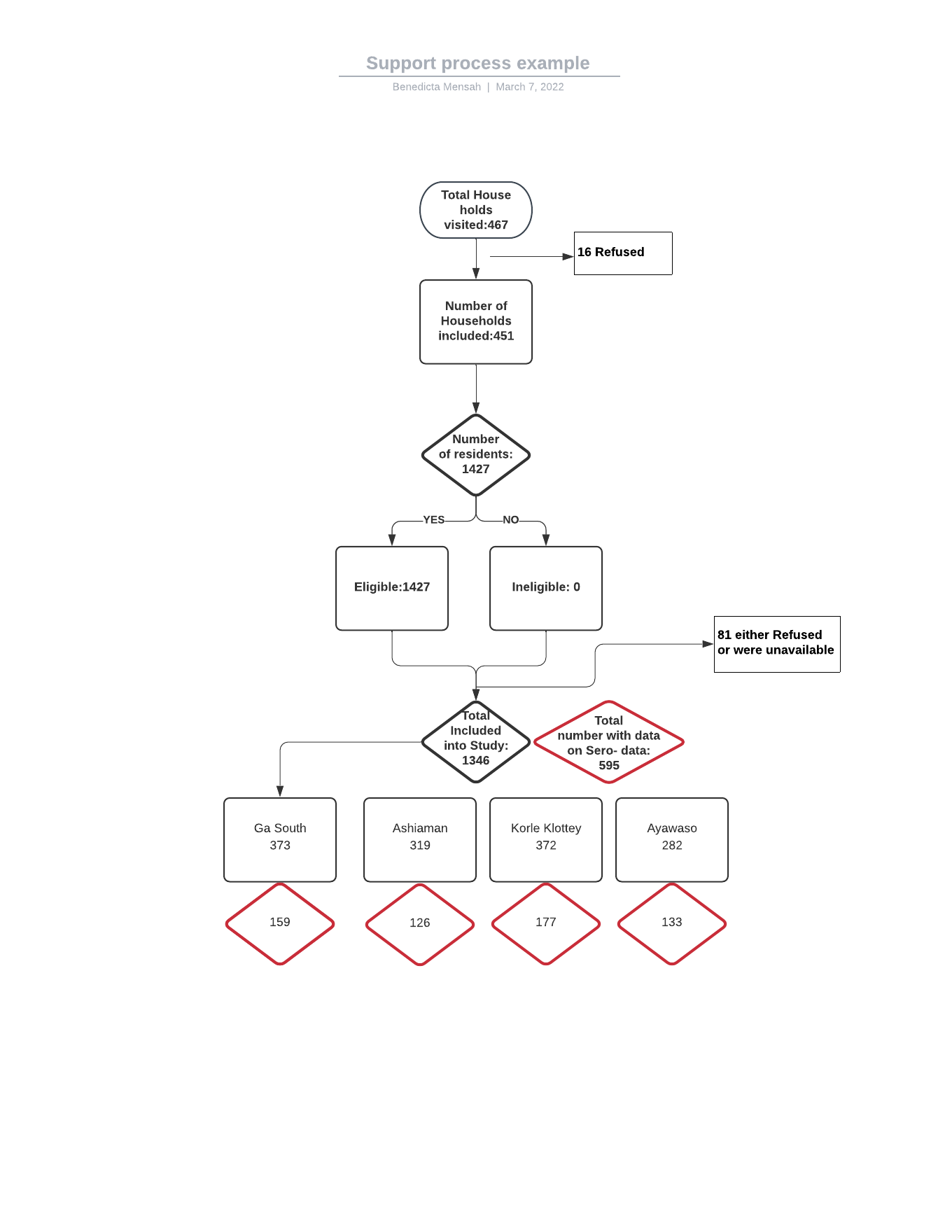


Figure S3. Description of the survey 3


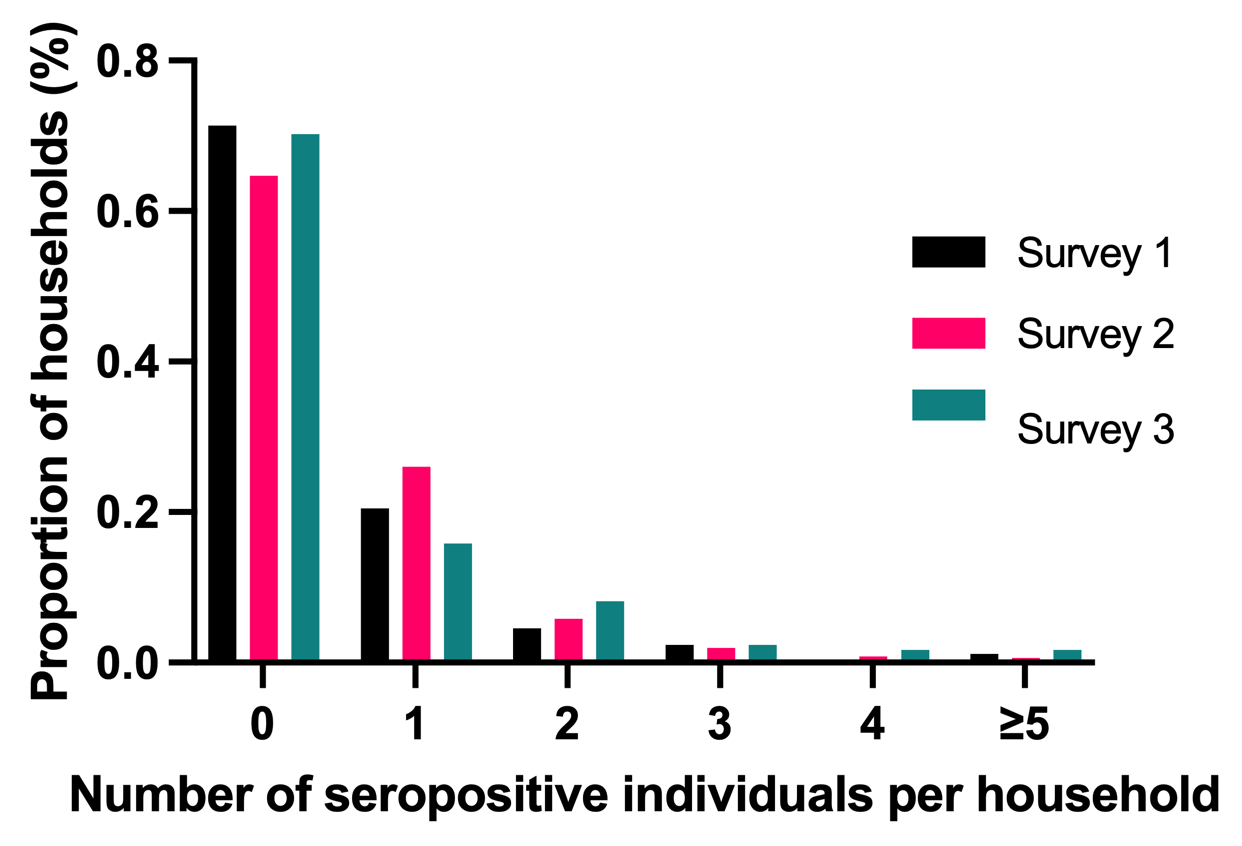


Figure S4. Proportion of Households and number of individuals who were seropositive for both Spike and Nucleocapsid

Sub. Table 1. Odds ratios of risk factors associated with Sars-Cov-2 seroprevalence in 3 consecutive surveys.

|  | **Survey1** | | | **Survey2** | | | **Survey3** | | |
| --- | --- | --- | --- | --- | --- | --- | --- | --- | --- |
| **Characteristics** | **OR** | **ci lower limit** | **Upper Limit** | **OR** | **ci lower limit** | **Upper Limit** | **OR** | **ci lower limit** | **Upper Limit** |
| **Age Group (years)** |  |  |  |  |  |  |  |  |  |
| 0-19 |  |  |  |  |  |  |  |  |  |
| 20-39 | 0.9 | 0.4 | 2 | 0.6 | 0.2 | 1.7 | 2.9* | 1.2 | 7.2 |
| 40 and above | 1.1 | 0.4 | 2.9 | 1 | 0.3 | 3 | 3.0* | 1.1 | 8.5 |
| **Gender** |  |  |  |  |  |  |  |  |  |
| Males |  |  |  |  |  |  |  |  |  |
| Females | 1.5 | 0.9 | 2.3 | 1.4 | 0.9 | 2.4 | 0.9 | 0.6 | 1.5 |
| **Marital Status** |  |  |  |  |  |  |  |  |  |
| Single |  |  |  |  |  |  |  |  |  |
| Maried/Union | 0.8 | 0.4 | 1.5 | 1.8 | 0.9 | 3.4 | 0.9 | 0.5 | 1.5 |
| Widowed | 1.2 | 0.4 | 3.8 | 1 | 0.3 | 3.3 | 0.9 | 0.3 | 2.7 |
| Divorced/separated | 1.9 | 0.7 | 5.6 | 1.8 | 0.5 | 7.3 | 1 | 0.2 | 4.3 |
| **Education Level** |  |  |  |  |  |  |  |  |  |
| Never been to school |  |  |  |  |  |  |  |  |  |
| Primary | 2 | 0.8 | 5 | 1.2 | 0.5 | 3 | 0.6 | 0.2 | 1.5 |
| Secondary | 1.9 | 0.8 | 4.4 | 1 | 0.4 | 2.3 | 0.6 | 0.3 | 1.3 |
| Tertiary | 2.3 | 0.9 | 6.1 | 0.7 | 0.3 | 1.9 | 0.5 | 0.2 | 1.2 |
| **Occupation** |  |  |  |  |  |  |  |  |  |
| None |  |  |  |  |  |  |  |  |  |
| Civil servant | 1.4 | 0.7 | 2.8 | 1.3 | 0.6 | 3.1 | 1.6 | 0.7 | 3.6 |
| Pupil/Student | 1.3 | 0.7 | 2.3 | 1.6 | 0.6 | 4.2 | 3.2* | 1.2 | 8.9 |
| Artisan | 1.2 | 0.3 | 4.9 | 0.3 | 0.03 | 2.5 | 0.7 | 0.1 | 3.2 |
| Other | 1.2 | 0.6 | 2.6 | 1.9 | 0.8 | 4.4 | 1 | 0.4 | 2.4 |
| **Symptoms score** |  |  |  |  |  |  |  |  |  |
| No symtpoms |  |  |  |  |  |  |  |  |  |
| One to two symptoms | 1.7 | 0.4 | 7.8 | 0.8 | 0.2 | 2.8 | 0.7 | 0.4 | 1.4 |
| 3 to 5 symptoms | 1.3 | 0.3 | 6 | 0.7 | 0.2 | 2.3 | 0.9 | 0.5 | 1.8 |
| more than 5 symptoms | 1.5 | 0.3 | 7.8 | 1.1 | 0.3 | 4.2 | 1.9 | 0.8 | 5 |
| **Hospitalized for any of these symptoms** |  |  |  |  |  |  |  |  |  |
| Yes |  |  |  |  |  |  |  |  |  |
| No | 0.4 | 0.1 | 1.2 | 1.7 | 0.5 | 5.6 | 0.7 | 0.3 | 1.7 |
| **Number of HH members** |  |  |  |  |  |  |  |  |  |
| 1-2 |  |  |  |  |  |  |  |  |  |
| 3-5 | 1 | 0.5 | 1.7 | 1.4 | 0.8 | 2.4 | 0.7 | 0.4 | 1.2 |
| >5 | 1 | 0.6 | 1.8 | 1.8 | 0.9 | 3.5 | 0.7 | 0.4 | 1.5 |
| **Have you been vaccinated?** |  |  |  |  |  |  |  |  |  |
| Yes |  |  |  |  |  |  |  |  |  |
| No |  |  |  | 1.2 | 0.5 | 2.9 |  |  |  |
